# Salt Shock Responses of *Microcystis* Revealed through Physiological, Transcript, and Metabolomic Analyses

**DOI:** 10.3390/toxins12030192

**Published:** 2020-03-18

**Authors:** Maxime Georges des Aulnois, Damien Réveillon, Elise Robert, Amandine Caruana, Enora Briand, Arthur Guljamow, Elke Dittmann, Zouher Amzil, Myriam Bormans

**Affiliations:** 1 IFREMER-Phycotoxins Laboratory, IFREMER, F-44311 Nantes, France; Damien.Reveillon@ifremer.fr (D.R.); elise.robert@ifremer.fr (E.R.); amandine.caruana@ifremer.fr (A.C.); enora.briand@ifremer.fr (E.B.); zouher.amzil@ifremer.fr (Z.A.); 2 Department of Microbiology, Institute for Biochemistry and Biology, University of Potsdam, Karl-Liebknecht-Str. 24/25, 14476 Potsdam, Germany; guljamow@uni-potsdam.de (A.G.); editt@uni-potsdam.de (E.D.); 3 UMR CNRS 6553 ECOBIO, University of Rennes, 35042 Rennes, France; myriam.bormans@univ-rennes1.fr

**Keywords:** *Microcystis aeruginosa*, microcystin, salt stress, metabolomic, transcript

## Abstract

The transfer of *Microcystis aeruginosa* from freshwater to estuaries has been described worldwide and salinity is reported as the main factor controlling the expansion of *M. aeruginosa* to coastal environments. Analyzing the expression levels of targeted genes and employing both targeted and non-targeted metabolomic approaches, this study investigated the effect of a sudden salt increase on the physiological and metabolic responses of two toxic *M. aeruginosa* strains separately isolated from fresh and brackish waters, respectively, PCC 7820 and 7806. Supported by differences in gene expressions and metabolic profiles, salt tolerance was found to be strain specific. An increase in salinity decreased the growth of *M. aeruginosa* with a lesser impact on the brackish strain. The production of intracellular microcystin variants in response to salt stress correlated well to the growth rate for both strains. Furthermore, the release of microcystins into the surrounding medium only occurred at the highest salinity treatment when cell lysis occurred. This study suggests that the physiological responses of *M. aeruginosa* involve the accumulation of common metabolites but that the intraspecific salt tolerance is based on the accumulation of specific metabolites. While one of these was determined to be sucrose, many others remain to be identified. Taken together, these results provide evidence that *M. aeruginosa* is relatively salt tolerant in the mesohaline zone and microcystin (MC) release only occurs when the capacity of the cells to deal with salt increase is exceeded.

## 1. Introduction

The cyanobacterial bloom-forming genus *Microcystis* is described as one of the most widespread genera in freshwater ecosystems [1]. In addition, *Microcystis* has been particularly well studied because of its ability to produce the hepatotoxin microcystin (MC). Indeed, with nearly 279 MC variants described in the literature [2], the occurrence of MCs in aquatic ecosystems represents a threat to both human and animal health [3]. Within the *Microcystis* genus, the species *M. aeruginosa* has received particular attention, being the dominant species in blooms worldwide [1,4]. 

Several studies have shown that *M. aeruginosa’s* influence is extending to coastal ecosystems [5] and it is described as the dominant species in brackish water, e.g., in some part of the Baltic sea [6,7]. The first occurrence of MCs in coastal environment was revealed by the detection of MCs in marine mussels, but no MC-producing organism was clearly identified [8]. Although some marine and estuarine cyanobacteria such as *Leptolyngbya*, *Oscillatoria*, and *Synechococcus* have been described as MC producers [9], the occurrence of MCs in coastal environments is mostly derived from the transfer of MC-producing cyanobacteria through the freshwater-to-marine continuum [5,10]. As a consequence, the accumulation of transferred MCs was described in marine fauna such as sea otters and shellfish [11,12,13]. The occurrence of *M. aeruginosa* and/or MCs in brackish waters was reported in many locations in the United States, South America, Australia, Europe including France, Japan, or India [14,15,16,17,18,19,20] and even became recurrent in San Francisco Bay, USA [21] and in the Patos Lagoon, Brazil [22]. Long-term survey and model predictions pointed out the positive impact of climate change on the intensity and frequency of this phenomenon through the intensification of precipitation and longer drought periods [15,16,23,24]. The fate of *M. aeruginosa* and the production of MCs during this transfer raised the question of how *M. aeruginosa* cells would deal with the sudden salt variations they are likely to encounter along the way. 

In 1985, Reed and Walsby defined *Microcystis* as a very salt-sensitive genus [25]. Subsequently, several studies showed that the growth rate of *Microcystis* remained unaffected by salt addition up to a salinity of 10 [26,27]. Based on field and laboratory experiments, *M. aeruginosa* salt tolerance now ranges between 0 and 18 [27,28,29,30,31]. Salinity variation may affect cyanobacteria physiology through osmotic and ionic stresses, thus disturbing the cellular osmotic balance [32]. When salt stress overcomes cell salt tolerance, salt stress enhances the production of reactive oxygen species and induces programmed cell death in *M. aeruginosa*, resulting ultimately in cell lysis [29,33]. Within the range of salt tolerance of freshwater cyanobacteria, a salt shock may decrease the growth rate [27] and impact the photosynthetic activity of photosystem II [29,34]. 

The impact of salt variation on MC production by *M. aeruginosa* has led to contrasting results such as an increase in MC cellular quotas [35], or a decrease in intracellular MC content and MC production [27,36], or no effect [18]. When the salt tolerance of the cell was exceeded, cell lysis occurred resulting in MC release into the surrounding medium [29,31]. Moreover, the majority of these studies quantified the MCs as MC-LR equivalent and did not consider the response of several variants to salinity stress. 

The main physiological response of cyanobacteria to cope with salinity variation is the accumulation of compatible solutes to sustain turgor pressure and the osmotic balance of cells [32]. This physiological trait in *M. aeruginosa* is still overlooked, despite the presence of *M. aeruginosa* in estuaries and its apparent tolerance up to the mesohaline zone. After the report of glucosylglycerol accumulation [37], sucrose was identified in the particularly salt-tolerant *M. aeruginosa* strain PCC 7806 [38] and further confirmed in the field where the occurrence of the “sucrose” gene was correlated with the brackish origin of *M. aeruginosa* [18]. Hence, the accumulation of several compatible solutes could explain the differences in salt tolerance among *M. aeruginosa* strains [18,30,39]. 

In order to better characterize the mechanisms involved in *M. aeruginosa* cells coping with a sudden increase in salinity, physiological, transcriptomic, and metabolic responses of two strains of *M. aeruginosa*, which produce different MC variants and were isolated from fresh water (PCC 7820) and brackish (PCC 7806) environments, were monitored at different time scales. The physiological responses over 7 days were studied through monitoring of the photosynthetic activity and cellular growth. Short-term stress (<2 h) was investigated by RT-qPCR, targeting (i) two genes coding for stress biomarkers, superoxide dismutase (*sod*) and chaperonine (*groEL*), (ii) two genes coding for proteins belonging to photosystem I (PSI) and photosystem II (PSII) (*psaA* and *psbC*), (iii) as well as two genes involved in sucrose synthesis (*sppA* and *spsA*). In addition, the production of several MC variants (both intracellular and extracellular) was investigated by a targeted analysis based on liquid chromatography coupled to tandem mass spectrometry. Untargeted metabolomic analysis was also conducted in order to study the dynamics of the metabolome and to identify putative biomarkers of salt stress in the two *M. aeruginosa* strains. As both strains were isolated from two different salinity environments (i.e., fresh and brackish waters) and produced distinct MC variants, we expected different responses involving specific cellular, molecular, and metabolic mechanisms.

## 2. Results

### 2.1. Growth of M. aeruginosa during a Salt Shock

**Figure 1:**
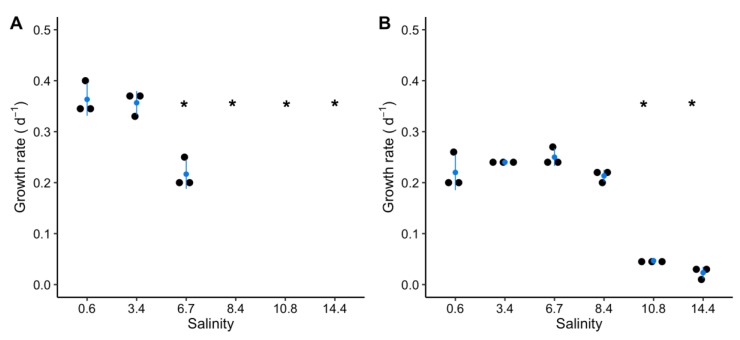
Growth rates for *Microcystis aeruginosa* Pasteur Culture Collection of Cyanobacteria (PCC) 7820 (freshwater) (**A**) and PCC 7806 (brackish water) (**B**) for each salt treatment. The absence of data represents negative growth rates. Blue dots represent the mean and blue error bars represent the standard deviation of the three replicates (black dots). Significant differences are indicated by * between the control and each condition (Dunnett’s test, *p* < 0.05).

For both strains, the calculated growth rates significantly decreased when salinity increased (one-way ANOVA, *p* < 0.05) (Figure 1 and Figure S1). Indeed, at salinities 6.7 and above, growth rates of PCC 7820 significantly decreased compared to the control (Dunnett’s test, *p* < 0.05) (Figure 1A and Figure S1A). Concerning PCC 7806 the growth rate significantly decreased only for salinities of 10.8 and 14.4 compared to the control (Dunnett’s test, *p* < 0.05) (Figure 1B and Figure S1B).

### 2.2. Photosynthetic Activity

The photosynthetic activity was monitored using the maximum quantum yield of PSII (F_V_/F_M_) as a proxy of the physiological state.

**Figure 2:**
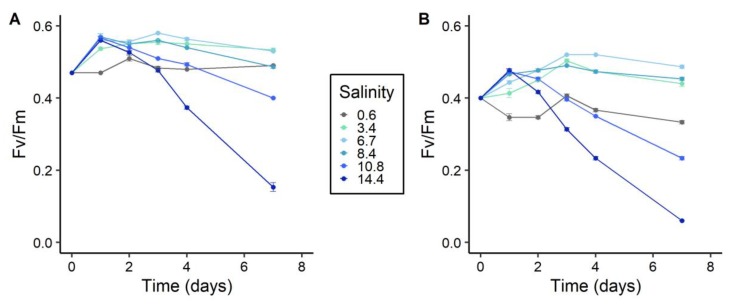
Maximum quantum yield of the photosystem II (PSII) (F_V_/F_M_) over time after salt treatment for *M. aeruginosa* PCC 7820 (freshwater) (**A**) and PCC 7806 (brackish water) (**B**). Triplicates of culture are represented as a mean and error bars represent the standard deviations (*n* = 3).

For both strains, the F_V_/F_M_ values remained stable in the control condition around 0.47 and 0.37, respectively (Figure 2). For all salt-treated cultures, an increase in the F_V_/F_M_ values was obtained within the first 24 h after the salt addition. Afterward, the F_V_/F_M_ values remained stable for salinities between 3.4 and 8.4 at ca. 0.48 and 0.55 for PCC 7820 and PCC 7806, respectively. However, F_V_/F_M_ values decreased from day 2 to day 7 for the highest salinities of 10.8 and 14.4. At the end of the experiment, the F_V_/F_M_ values for PCC 7820 and PCC 7806 were 0.15 and 0.06, respectively (Figure 2).

### 2.3. Relative Gene Expressions

**Figure 3:**
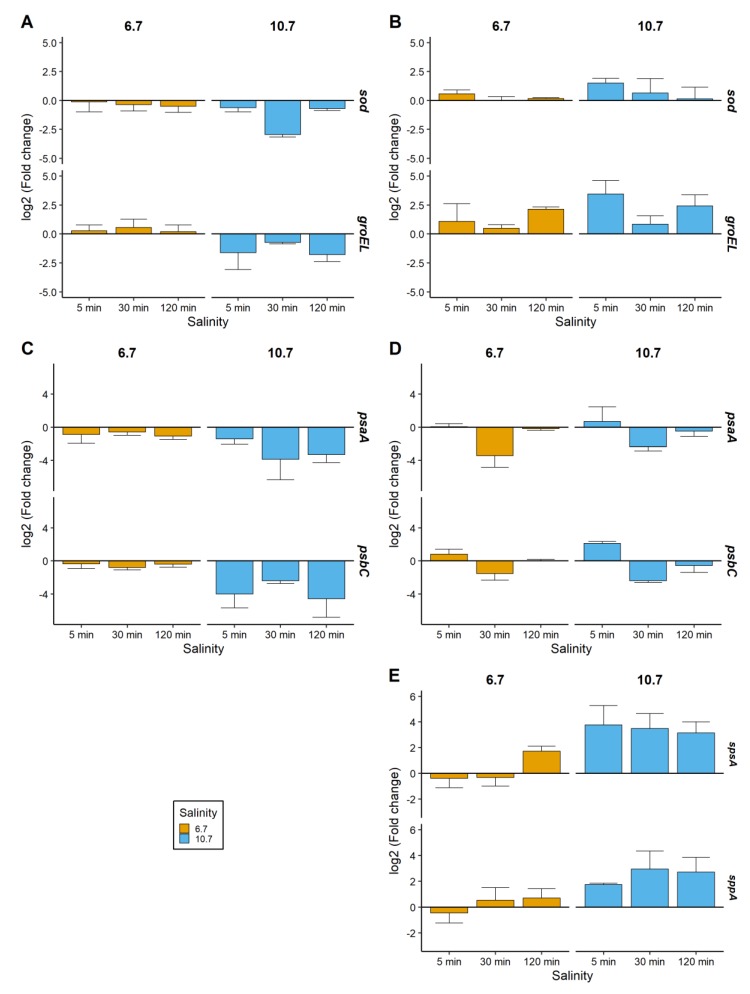
Effect of salt addition on the relative gene expressions expressed as log_2_(fold change). (**A**,**C**) Relative expressions in *M. aeruginosa* PCC 7820 (freshwater) for *sod*/*groEL* and *psaA*/*psbC* genes, respectively (**B**,**D**,**E**) Relative expressions in *M. aeruginosa* PCC 7806 (brackish water) of *sod*/*groEL*, *psaA*/*psbC*, and *spsA*/*sppA* genes. Gene expressions were normalized to the control condition for each sampling time. Triplicates of culture are represented as a mean and error bars represent the standard deviations (*n* = 3).

For *M. aeruginosa* PCC 7820 at salinity 6.7, the relative *sod*, *groEL, psaA*, and *psbC* gene expressions remained in the range of −1 to 1 (Figure 3A,C). At salinity 10.7, they were downregulated, after 30 min for *sod* and after 5 and 120 min for *groEL* (Figure 3A), and at all sampling times for *psaA* and *psbC* (Figure 3C).

For *M. aeruginosa* PCC 7806, the *sod* expression at salinity 6.7 was within 0 and 1 at each sampling time (Figure 3B). However, at salinity 10.7, the *sod* mean expression (±standard deviation) was upregulated 1.5 (±0.39) after 5 min and then decreased between 0 and 1 (Figure 3B). The *groEL* gene expression at salinity 10.7 was upregulated after 5 and 120 min with log_2_(fold change) values of 3.5 (±1.2) and 2.4 (±0.95), respectively (Figure 3B). At salinities 6.7 and 10.8, the *psaA* expression was downregulated after 30 min and remained similar to the control condition after 120 min (Figure 3D). The *psbC* expression remained between 0 and 1 at salinity 6.7 while at a salinity of 10.7, it started an upregulation after 5 min, then decreased after 30 min, and remained similar to the control condition after 120 min (Figure 3D). In PCC 7806, the *spsA* and *sppA* relative gene expressions after salt addition were upregulated after 120 min at salinity 6.7 and at each sampling time at a salinity of 10.7 (Figure 3E). However, no amplification product was found in PCC 7820 using the primer pairs designed for this study.

### 2.4. Metabolome Dynamics

**Figure 4:**
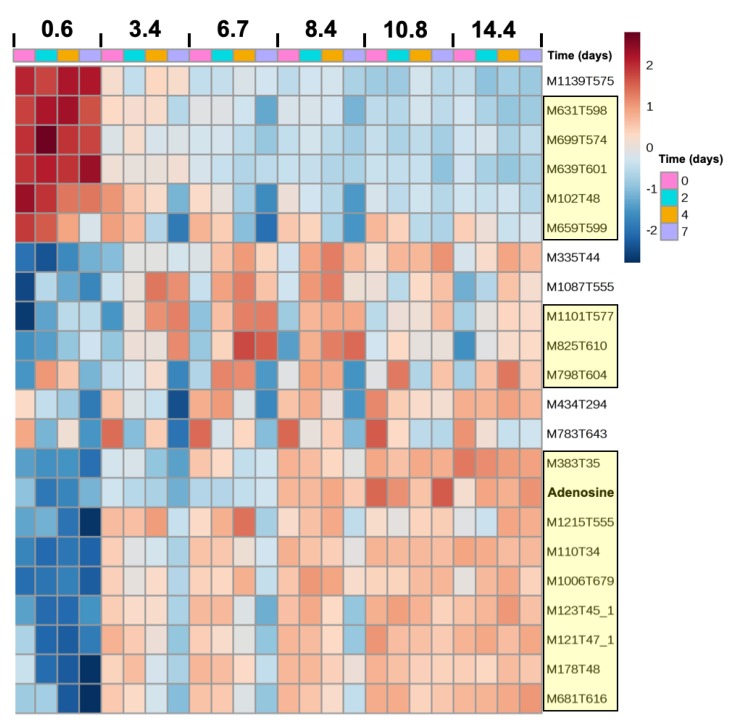
Heatmap of significantly salt-affected metabolites of *M. aeruginosa* PCC 7820 (freshwater). Metabolites highlighted in yellow are common to both strains. Data represented are means of triplicate. (M: *m/z*; T: retention time in seconds) and corresponded to features with a relatively lower (blue) and higher (red) area.

**Figure 5:**
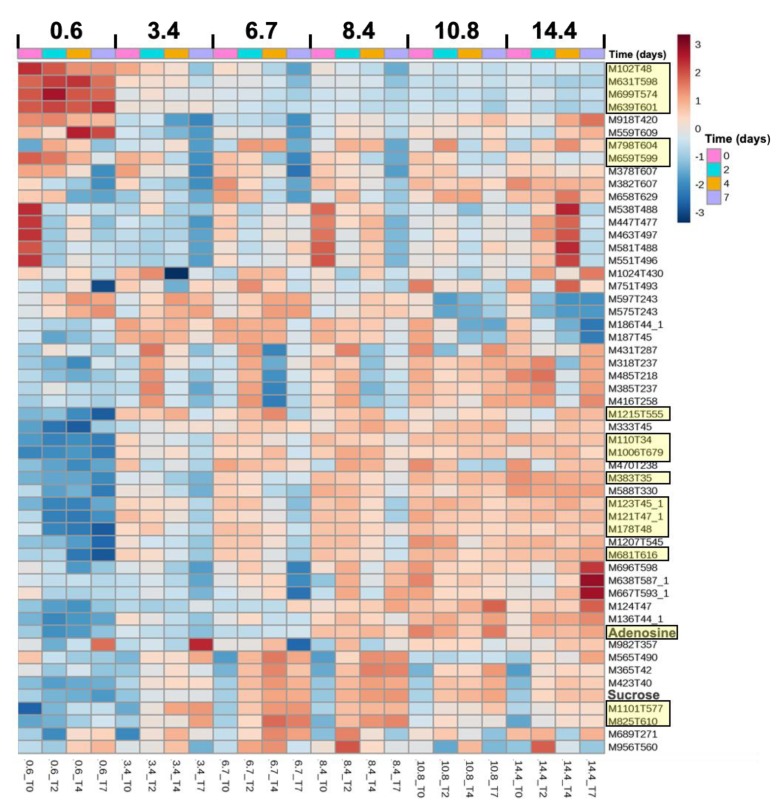
Heatmap of significantly salt-affected (salt and salt–time interaction) metabolites of *M. aeruginosa* PCC 7806 (brackish water). Metabolites highlighted in yellow are common to both strains. Data represented are means of triplicate. (M: *m/z*; T: retention time in seconds) and corresponded to features with a relatively lower (blue) and higher (red) area.

The effects of salt, time, and their interaction were investigated on the 382 metabolites obtained by the metabolomic analysis. While salt and time submodels of the ANOVA-simultaneous component analysis (ASCA) were validated by a permutation test for both strains, the salt–time interaction submodel was only validated for *M. aeruginosa* PCC 7806 metabolomic dataset (*p* = 0.41 for PCC 7820). The selection of significant features relative to salt, time, and their interaction was performed based on the squared prediction error (SPE) and leverage scores [40]. In PCC 7820, 22 (Figure 4) and 42 metabolites (Figure S2) were significantly affected by salt and time factors, respectively. In PCC 7806, 32 (Figure 5), 34 (Figure S3), and 29 metabolites (Figure 5) were significantly affected by salt, time, and interaction factors, respectively.

The heatmaps showing the relative abundances of all significantly salt-affected metabolites for PCC 7820 (Figure 4) and PCC 7806 (Figure 5) revealed that both specific and 17 common metabolites (highlighted in yellow in the figures) were impacted by salt (either under- or over-accumulated). Relative expression patterns of the 17 common metabolites were similar in the two strains in response to salt and time (Figure 4 and Figure 5) with 12 metabolites accumulated at higher salinities and 5 metabolites accumulated at lower salinities. PCC 7806 showed the greatest diversity of metabolites impacted by salt treatment. The mass and retention time of all these metabolites are provided in Table S2. Among these metabolites, two compounds were successfully identified.

Adenosine was identified in both strains, based on the similarity of HRMS/MS spectra compared to the GNPS (Global Natural Product Social Molecular Networking) database (i.e., 8 MS/MS peaks were shared and the similarity or cosine score was 0.99 while a value of 1 indicates identical spectra).

Sucrose was identified in PCC 7806, and its abundance was significantly impacted by the interaction of salt and time according to the ASCA submodel. Gas chromatography-flame ionization detector (GC-FID) analysis confirmed that sucrose was not detected in the control condition (i.e., below the detection limit), whereas it was quantified after 2 and 4 days of exposure at a salinity of 6.7 and 10.7. Accordingly, the accumulation of sucrose in PCC 7806 cells ranged between 50 and 150 fg/cell (Figure S4). By contrast, sucrose was neither detected in the metabolomic dataset nor by GC-FID in PCC 7820. While trehalose was identified in PCC 7820 but it was not significantly impacted by salt, time, or their interaction according to ASCA analysis.

### 2.5. MC Cellular Quotas

**Figure 6:**
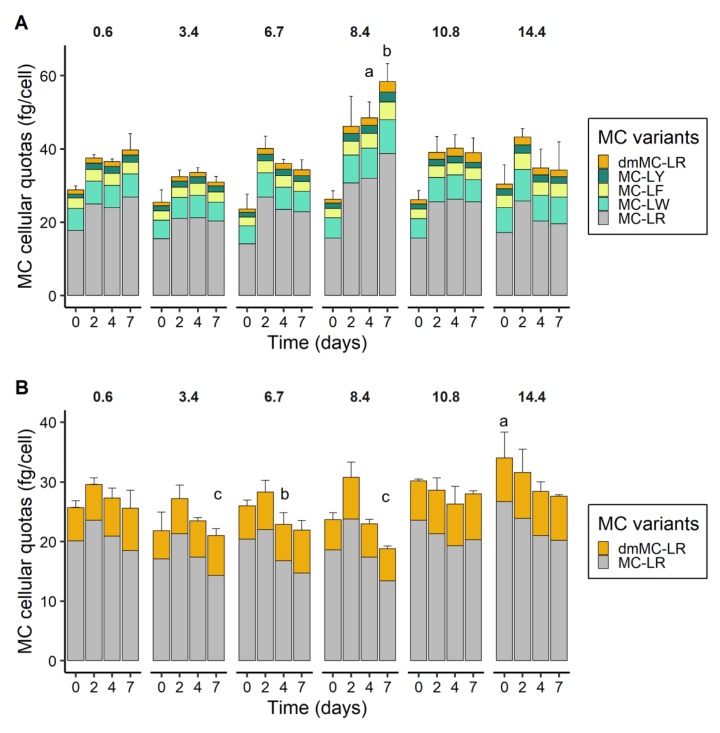
Microcystin cellular quotas over time and salinities for *M. aeruginosa* PCC 7820 (freshwater) (**A**) and PCC 7806 (brackish water) (**B**). Error bars represent the standard deviations (*n* = 3). Letters indicate significant differences compared to the control condition (one-way ANOVAs and Dunnett’s test on salinity treatments at each respective time point).

Five MC variants were quantified in *M. aeruginosa* PCC 7820 with the following decreasing order of cellular quotas: MC-LR (14–39 fg/cell) > MC-LW (4.9–9.3 fg/cell) > MC-LF (2.4–4.8 fg/cell) > MC-LY (1.1–2.7 fg/cell) and dmMC-LR (0.90–2.9 fg/cell) (Figure 6A).

Two MC variants (MC-LR and dmMC-LR) were quantified in *M. aeruginosa* PCC 7806 with cellular quotas of 13–27 fg/cell for MC-LR and 4.7–7.7 fg/cell for dmMC-LR (Figure 6B).

For PCC 7820, statistically significant effects of salinity were obtained only on days 4 and 7 between the control condition and a salinity of 8.4 (Figure 6A). For PCC 7806, the total MC cellular quota was significantly different from the control on day 0 at salinity 14.4, on day 4 at salinity 6.7, and on day 7 at salinities 3.4 and 8.4.

The relative contribution of each MC variant to the total MC profiles revealed a relative stability of MC ratios over time and salinities for both strains (Figure S5). Irrespective of salinity and time, MC variant proportions for *M. aeruginosa* PCC 7820 represented 60%, 20%, 10%, 5%, 5%for MC-LR/-LW/-LF/-LY/dmMC-LR respectively (Figure S5A). Concerning, *M. aeruginosa* PCC 7806, MC-LR/dmMC-LR proportions represented 75%, 25%of the MC profile (Figure S5B).

**Figure 7:**
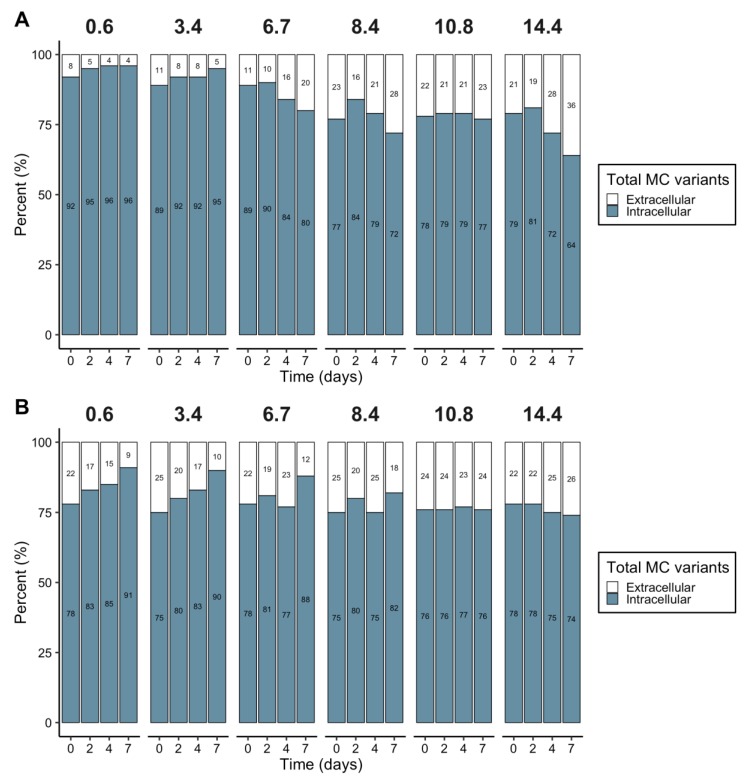
Intracellular and extracellular microcystin (MC) proportions over time for each salinity for *M. aeruginosa* PCC 7820 (freshwater) (**A**) and PCC 7806 (brackish water) (**B**).

The proportion of extracellular MCs in *M. aeruginosa* PCC 7820 decreased in the control and at salinity 3.4, from 8.4% ± 0.90% to 3.7% ± 0.50% and 11% ± 2.4% to 5.2% ± 0.30% between 0 and 7 days, respectively (Figure 7A). At higher salinities, the proportion of MCs in the medium increased with both salinities and time and the maximum was reached at salinity 14.4, with an increase from 21% ± 1.9% to 36% ± 2.4% between 0 and 7 days (Figure 7A). In the strain *M. aeruginosa* PCC 7806, the proportion of extracellular MCs decreased over time at salinities below 8.4. At a salinity of 10.8, the extracellular MC fraction remained stable at 24% ± 1.1%. For the highest salinity, a slight increase in extracellular MCs was noted from 22% ± 3.0% to 26% ± 2.6% (Figure 7B). The extracellular MC concentrations, expressed in ng/mL of culture, are provided in Figure S6.

The MC production rate was calculated for all MC variants over the growth period and plotted against the calculated growth rate over the same period. For each variant and both strains, the growth rate and MC production rate were significantly correlated according to the Pearson correlation test (*p* < 0.05) (Figure S7).

## 3. Discussion

The impact of a salt shock was investigated using physiological and omics approaches on two strains of *M. aeruginosa* isolated from contrasting environments, namely fresh and brackish waters for PCC 7820 and PCC 7806, respectively. The main consequence of salt stress, for both strains, was first a decrease of photosynthetic activity, as a precursor of the salinity-dependent decreased growth. Hence, the photosynthetic activity of both strains, was negatively impacted, as a consequence of the fast downregulation of *psaA* and *psbC* genes that encode proteins belonging to PSI and PSII, respectively. These results confirmed the impact of salts on the photosynthetic activity of cyanobacteria such as *M. aeruginosa* [29,41]. The downregulation of *psaA* and *psbC* genes could be the consequence of osmotic disturbance. Indeed, Allakhverdiev and Murata (2008) showed that NaCl addition was responsible for the accumulation of Na^+^ in the cytosol, which resulted in extrinsic protein dissociation from the photosystems and ultimately a decrease of photosystem activity. The decrease in Fv/Fm values also suggested perturbations of the photosynthetic activity in the cells, which could be related to a change of photosystem stoichiometry (i.e., ratio of PSI/PSII) in response to salt stress [34,41,42]. Although the physiological state of both strains was negatively influenced by the salt shock, their growth was differentially impacted. Above a salinity of 3.4, the growth rate of PCC 7820 significantly decreased and became negative above 6.7, evidencing cell lysis. The strain PCC 7806 exhibited a higher salt tolerance (up to 8.4) with no negative growth rate at higher salinity conditions. Hence, as previously observed, these two strains exhibited distinct salt tolerances, with the freshwater PCC 7820 strain less tolerant compared to the brackish PCC 7806 one [30].

Intraspecific variability in response to salt addition was also observed at the gene expression level. As the addition of salt may be responsible for the induction of oxidative stress [29], the *sod* gene, coding for an antioxidant enzyme and the *groEL* gene, involved in the oxidative stress response, were monitored. An upregulation of both genes only occurred in the more salt-tolerant strain *M. aeruginosa* PCC 7806. These genes have been already shown to be overexpressed in *M. aeruginosa* in response to sulfate addition for *sod* [43] or in the salt-tolerant genus *Synechocystis* [44], and in response to limiting growth conditions for *groEL* [45]. As discussed by Schuurmans et al. [46] with oxidative stress, the higher salt tolerance of PCC 7806 could be due to an earlier response of stress marker genes that protect this strain at higher salinities.

Altogether, these studies including the present one showed intraspecific differences in salt stress responses in *M. aeruginosa*, which could be related to the origin of the strains [47] and specific adaptation involving molecular mechanisms. Such mechanisms include the acquisition of specific genes through horizontal gene transfer [48,49], mutations and genomic rearrangements via the activity of transposable elements [50], or epigenetic processes [51]. The understanding of such regulatory molecular mechanisms and their environmental drivers such as salt variation will represent an important step toward predicting or estimating cyanobacterial capability to cope with salt variation and to expand their development in coastal environments.

The metabolomic approach represents a useful tool to study the cellular response to salt stress. Indeed, metabolomics provide a metabolic fingerprint showing the ultimate salt response of *M. aeruginosa* by analyzing simultaneously the relative expression of hundreds of metabolites [52]. The metabolomes of both strains were impacted by salt addition, which suggests a modification of various cellular functions in salt-stress cells as reported by Hagemann [32]. In this experiment, it was possible to distinguish the metabolic fingerprint of the two strains by the relative abundances of some metabolites that were significantly impacted by salinity, time, and their interaction. Indeed, as for adenosine accumulation in response to increasing salinities, 16 other common metabolites were significantly impacted by salt stress in both strains. Therefore, and similarly to transcriptomics [44], this metabolomic approach highlighted putative biomarkers of salt stress. Unfortunately, the main challenge is the dereplication and identification of these metabolites [52]. A higher number of metabolites impacted by salt stress were found in *M. aeruginosa* PCC 7806, which suggests that the higher salt tolerance of this strain is related to the accumulation of a greater diversity of compounds. In particular, as previously found in salt shock, the strain *M. aeruginosa* PCC 7806 synthesizes and accumulates sucrose in response to salt increase [38]. In the first minutes after the stress, an increase in *spsA* and *sppA* gene expressions was obtained evidencing that sucrose synthesis is a highly dynamic process after the onset of salt stress [32,38]. Interestingly, sucrose is not only accumulated in *M. aeruginosa* as a response to salt shock. Indeed, it also constitutes a long-term response in salt-tolerant strains as a key metabolite during salt variation [18,30]. Despite the fact that trehalose was identified in *M. aeruginosa* PCC 7820, it was not significantly accumulated in response to salt stress contrary to what we previously found with acclimated cultures [30]. Surprisingly, no lipids involved in membrane fluidity, such as monogalactosylglycerol, were significantly impacted by the salt shock experiment while several were found on both acclimated *M. aeruginosa* PCC 7820 and 7806 [30]. It may be assumed that an increase of unsaturated glycolipids is a marker of acclimation to brackish conditions in *M. aeruginosa*. Further investigations should be conducted to identify these metabolites and confirm that they are specific biomarkers of salt stress in *M aeruginosa*.

Under routine culture conditions, MCs remained mostly intracellular (that is, until cell lysis) and their production is considered to be constitutive [53]. Hence, variations in MC production and the associated cellular quotas are more likely related to the indirect impact of abiotic and biotic factors on cell growth [54]. This was confirmed here as the salt shock experiment also resulted in a reduction in the growth rate that was correlated to a reduction in MC production rate. Relatively stable MC cellular quotas (i.e., total and for each MC variant) were found irrespective of the salt treatments and *M. aeruginosa* strains, suggesting no particular selection of variants with an increased salinity. An indirect impact of salinity on MC production through a decrease of growth was also found in *M. aeruginosa* PCC 7820 and PCC 7806 acclimated to different salinities [30]. The release of MCs in the surrounding medium occurred when the salt tolerances of *M. aeruginosa* PCC 7820 and PCC 7806 were surpassed, as previously shown for the *M. aeruginosa* strain LB 2385 [29] and natural bloom samples [31].

Salt shock induced an oxidative stress in *M. aeruginosa* resulting in accumulation of reactive oxygen species and lipid peroxidation [29]. One of the suggested intracellular functions of MCs is to protect the cells against oxidative stress by binding to cysteine residues on redox sensitive proteins, and this was reported under high irradiance stress [55,56] and limiting growth conditions [45]. Nevertheless, this study did not find strong variations in MC-free fractions that could have been attributed to a protein binding process. Further investigations should be conducted on the MC-bound fraction during salinity variation. Together with other studies of salt impact on MC production [27,29,30,31], this laboratory study suggests that the toxicity of a *M. aeruginosa* bloom during a sudden transfer from a freshwater to a marine continuum, may not be increased by the induction of MC production. Altogether, our results suggested that salt treatment did not impact the synthesis and release of a specific MC variant. As some variants are known to be more toxic than others [3], this result therefore suggests no specific increase or decrease in toxicity potential for estuarine organisms. This assessment raises the question of the influence of a salt stress on the fitness of MC- and non-MC-producing genotypes as others environmental factors have been shown to drive their selection and consequently to explain toxicity variations during a natural bloom [31,57,58].

## 4. Materials and Methods

### 4.1. Organisms and Experimental Design

Two axenic MC-producing strains of *M. aeruginosa* from the Pasteur Culture (Paris, France) collection of Cyanobacteria (PCC; https://webext.pasteur.fr/cyanobacteria/) were studied, the PCC 7820 and PCC 7806. *M. aeruginosa* PCC 7820 was isolated in a freshwater lake in Scotland, while *M. aeruginosa* PCC 7806 was isolated in the brackish water of Braakmann Reservoir in Netherlands. Cells were routinely grown in modified BG11_0_ [59] supplemented with NaNO_3_ (2 mM) and NaHCO_3_ (10 mM), at constant temperature of 22 °C under a 12:12 h light:dark cycle using cool-white fluorescent tubes (Philips, Amsterdam, Netherlands) with 35 µmol photons m^−2^ s^−1^ illumination. Artificial sea water at a salinity of 36 was prepared with Milli-Q water and the addition of NaCl (450 mM), KCl (10 mM), CaCl_2_ (9 mM), MgCl_2_ (6H_2_O) (30 mM), and MgSO_4_ (7H_2_O) (16 mM) (Merck, Darmstadt, Germany) [60]. Selected salinities were obtained by dilutions with Milli-Q water before nutrient enrichment. Salinity was checked using a conductivity meter Cond 3110 Set 1 (WTW, Oberbayern, Germany). The salinity, as a non-dimensional number, was inferred from a measure of conductivity and temperature using the empirical relationship recommended by the United Nations Educational, Scientific and Cultural Organization (1985) [61]. Exponentially growing cells were transferred from BG11_0_ to fresh media in order to conduct salt shock experiments at five different salinities (3.4, 6.7, 8.4, 10.8, and 14.4) with a control condition in BG11_0_ at 0.6. Samples for cell enumeration and photosynthetic parameters were taken every day. Samples for MC and metabolomic analyses were taken at days (0, 2, 4, and 7).

Another set of experiments was conducted at salinities 0.6, 6.7, and 10.8 in the abovementioned conditions. After 5, 30, and 120 min, cell samples were collected for RNA extraction and transcript analyses of targeted genes.

### 4.2. Cell Enumeration and Photosynthetic Parameter

Cell enumeration was conducted by cytometry on fresh samples using an Accuri C6 flow cytometer (Becton Dickinson, Franklin Lakes, NJ, USA). Mean growth rates over 7 days were calculated using the least-squares regression method according to Wood et al. [62]. Before fluorescence analyses, samples (3 mL) were incubated in the dark for 15 min [63]. Maximum quantum efficiency of the photosystem II (F_V_/F_M_) was measured (455 nm, no DCMU addition and the basal fluorescence was not subtracted) every day using an Aquapen-C 100 fluorimeter (Photon Systems Instruments, Drasov, Czech Republic).

### 4.3. LC-MS/MS Analysis of MCs

Subsamples (15 mL) for LC-MS/MS analyses were obtained by centrifugation at 4248× *g* for 15 min at 4 °C. The supernatant was stored immediately at −80 °C and the remaining cell pellet was quenched in liquid nitrogen and stored at −80 °C until extraction. Cells and supernatants were extracted and analyzed as in Georges des Aulnois et al. [30]. Briefly, cells were extracted with MeOH using a mixer mill while extracellular MCs were concentrated by solid phase extraction on a C18 cartridge. MCs quantification was conducted by LC-MS/MS using an external calibration curve made with 9 standards (MC-RR, dmMC-RR, MC-LR, dmMC-LR, MC-LW, MC-LY, MC-LF, MC-LA, and MC-YR (Novakits, Nantes, France, purity >95%). The net production rate of each MC variant was calculated over 7 days according to Orr et al. [54].

### 4.4. RNA Isolation and RT-qPCR

RNA extraction was performed by adding 1 mL Trizol (Life Technologies, Carlsbad, CA, USA) and 200 µL chloroform to the cell pellets. Samples were then purified with RNA Clean-up XS MN kit (Macherey-Nagel, Düren, Germany) following the manufacturer’s instructions. RNA purity and concentration were checked using a Nanodrop ND-1000 spectrophotometer (Thermo Fischer Scientific, Waltham, MA, USA) at 260 and 280 nm. Purified extracts of RNA were treated with DNase (RNase-Free DNase Set, Quiagen, Hilden, Germany). RNA quality was checked on gel before reverse transcription using qScript Supermix (QuantaBio, Beverly, MA, USA). For RT-qPCR, a LightCycler 480 (Roche Applied Science, Penzberg, Germany) in combination with a SYBR green-based detection system (DyNamo ColorFlash SYBR green qPCR Kit, Thermo Fischer Scientific, Waltham, MA, USA) was used. Specific primer pairs for *rnpB*, *sod*, *groEL*, *psaA*, *psbC*, *sppA*, *spsA* genes were designed (Table S1) and tested in PCRs prior to RT-qPCR. The RNase P encoding gene *rnpB* was used as housekeeping gene for standardization, as described previously by Makower et al. [64]. Each RT-qPCR was carried out in three technical replicates. Raw data were converted using the software LC480Converter and primer efficiencies were calculated using LinRegPCR. Relative expression of targeted genes was calculated following the Pfaffl [65] method and expressed as log_2_(fold change). This method considers corrections for actual PCR efficiencies for each gene normalized to *rnpB* expression level. Each expression level was normalized to the gene expression found in the control condition for each sampling time.

### 4.5. Metabolomic Analyses

Metabolomic analyses were conducted on intracellular extracts prepared for MC analyses by ultra-performance liquid chromatography—high-resolution mass spectrometry (UPLC-HRMS) as described in Georges des Aulnois et al. [30]. Pool samples (QC) were prepared and injected ten times at the beginning of the batch sequence and then every ten samples (including blank). Blanks were prepared as cell pellets (i.e., MeOH and glass beads in a polypropylene tube). Data were deposited on DATAREF (https://doi.org/10.12770/b8a87122-4e4b-4d8a-9c24-a9059f6b49e3). To identify specific compounds of interest, tandem mass spectrometry analyses were carried out as in Georges des Aulnois et al. [30]. Assignation was conducted firstly with exact masses and freely available databases (e.g., HMDB, LipidMaps, Metlin, KEGG, http://ceumass.eps.uspceu.es/, Gil-de-la-Fuente et al. [66]), then with fragmentation patterns and using a molecular networking approach (https://gnps.ucsd.edu/, Wang et al. [67]). Acquisition and data processing were performed using Mass Hunter Workstation software (version B.06.01 and B.07, Agilent, Santa Clara, CAL, USA).

LC-HRMS raw data (.d) were converted to .mzXML format using MS-Convert (ProteoWizard 3.0, [68] and pre-processed with the Workflow4Metabolomics (W4M; http://workflow4metabolomics.org) e-infrastructure [69].

Peak picking, grouping, retention time correction, and peak filling were performed with the “centWave”, “density”, “obiwarp”, and “chrom” methods. Annotation (isotopes, adducts) was conducted with the “CAMERA” algorithm [70]. Intra-batch signal intensity drift was corrected by fitting a locally quadratic (loess) regression model to the QC values [71,72].

Three successive filtering steps using in-house scripts on R were applied to remove (i) variables with low intensities (exclusion of variables with signal to noise ration <10 compared to a blank), (ii) signals showing high variability (exclusion of variables with a coefficient of variation >25% in QC samples), and (iii) to suppress redundancy (exclusion of all variables but the most intense one when the coefficient of autocorrelation >80% at the same retention time). Pre-processing led to 2313 variables and 382 variables remained after the filtrations on which statistical analyses were performed.

To confirm the identification of sucrose and provide quantitative data, a GC-FID method (gas chromatography coupled to a flame ionization detector) was implemented, after the derivatization procedures described by Adams et al. (1999) [73].

### 4.6. Statistical Analyses

Statistical analyses were performed using R software (version 3.5.1) [74]. Data were presented as mean and standard deviation (SD). Homoscedasticity and normal distribution of residuals were checked using Bartlett and Shapiro–Wilk tests. Then, the effect of salinity on growth was tested using one-way analysis of variance (ANOVA) and the post-hoc Dunnett’s test was applied to check difference between growth rates compared to the control.

As the experimental design involved repeated measures on the same cultures in response to salt treatments, the statistical analyses on metabolomic data were performed using the “Time-series/Two-factor” module provided by MetaboAnalyst 4.0 [75]. Before statistical analysis, data were filtered as described above, normalized to the cell concentration, and log transformed. A Pareto scaling was applied as suggested by van den Berg et al. [76]. Effects of time, salinity, and their interaction were tested by applying an ANOVA-simultaneous component analysis (ASCA). ASCA is a generalization of ANOVA for univariate data to the multivariate case and adapted for omics data (i.e., the number of variables exceeds the number of experiments) [77]. Briefly, the ASCA approach decomposes the overall data variance into individual variances induced by salinity, time, as well as by their interaction for all metabolites. It then applies a principal component analysis (PCA) for each factor to summarize major variations as described in Nueda et al. [40]. Then the selection of metabolites that are closely following the detected trend as well as those that clearly diverge from the trend was conducted by the use of two statistics, the leverage and the squared prediction error (SPE) [40]. The leverage is a measure of the importance of the metabolites in the PCA model and SPE is a measure of the goodness of fit of the model for that specific metabolite. Then, metabolites with high leverage and low SPE are considered a well-modeled compound in the PCA model, while those with a high leverage and high SPE are considered as outliers [40]. Both well-modeled and outlier metabolites were then tentatively identified, as they represent putative biomarkers that respond to each factor. ASCA model validation was performed using a permutation test (100 permutations) according to Vis et al. [78].

## Supplementary Materials

The following are available online at https://www.mdpi.com/2072-6651/12/3/192/s1, Figure S1: Cell concentrations over time after salt treatments for *Microcystis aeruginosa* PCC 7820; Figure S2: Linear regression between growth rates and MC production rates for *M. aeruginosa* PCC 7820 (A) and PCC 7806 (B); Figure S3: Sucrose cellular quotas over time and salinities for *M. aeruginosa* PCC 7806; Figure S4. Heatmap of significantly time-affected metabolites of *M. aeruginosa* PCC 7820; Figure S5: Heatmap of significantly time-affected metabolites of *M. aeruginosa* PCC 7806; Figure S6. MC cellular quotas over time and salinities expressed as the percentage of each variant relative to the total amount of MCs per cell for *M. aeruginosa* PCC 7820 (A) and PCC 7806 (B); Figure S7. Linear regression between growth rates and MC production rates for *M. aeruginosa* PCC 7820 (freshwater) (A) and PCC 7806 (brackish water) (B). Table S1: Primer sequences used for RT-qPCR reactions, Table S2: Exact mass and retention time for metabolites impacted by salt factor.
