# Supplementary material for: Salt Shock Responses of Microcystis Revealed through Physiological, Transcript, and Metabolomic Analyses
Source: Toxins (Basel). 2020 Mar 18;12(3):192. doi: 10.3390/toxins12030192 (PMC7150857; doi:10.3390/toxins12030192)
Supplement: Supplementary file 1 [file toxins-12-00192-s001.pdf]

Supplementary Materials: Salt Shock Responses of *Microcystis* Revealed through Physiological, Transcript, and Metabolomic Analyses

Maxime Georges des Aulnois, Damien Réveillon, Elise Robert, Amandine Caruana, Enora Briand, Arthur Guljamow, Elke Dittmann, Zouher Amzil and Myriam Bormans

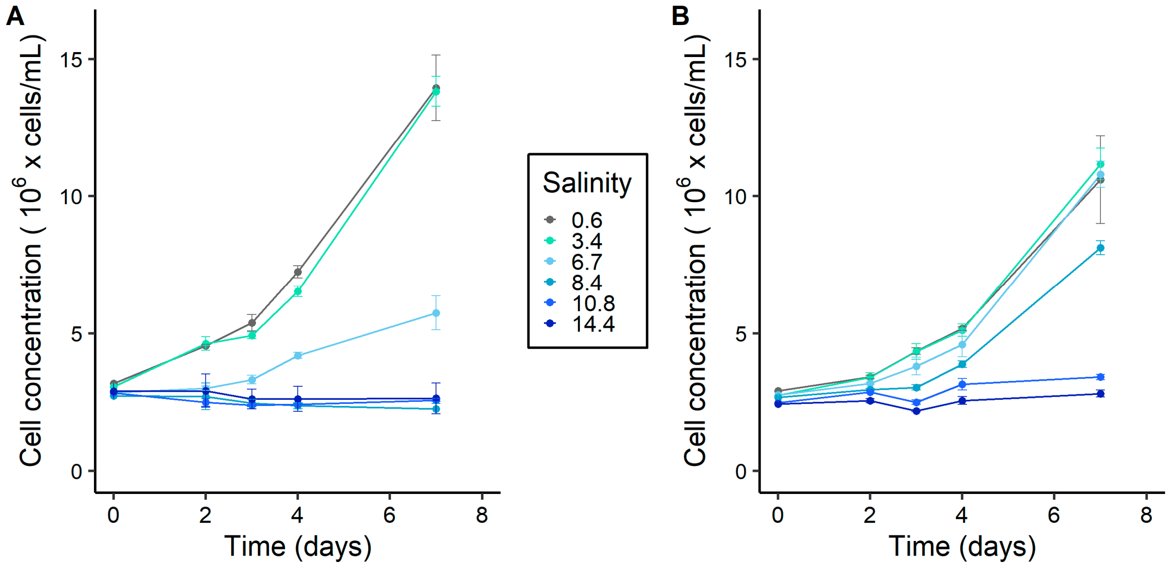

Figure S1. Cell concentrations over time after salt treatments for *Microcystis aeruginosa* PCC 7820 (freshwater) (**A**) and PCC 7806 (brackish water) (**B**). Triplicates of culture are represented as mean and error bars represent the standard deviation.

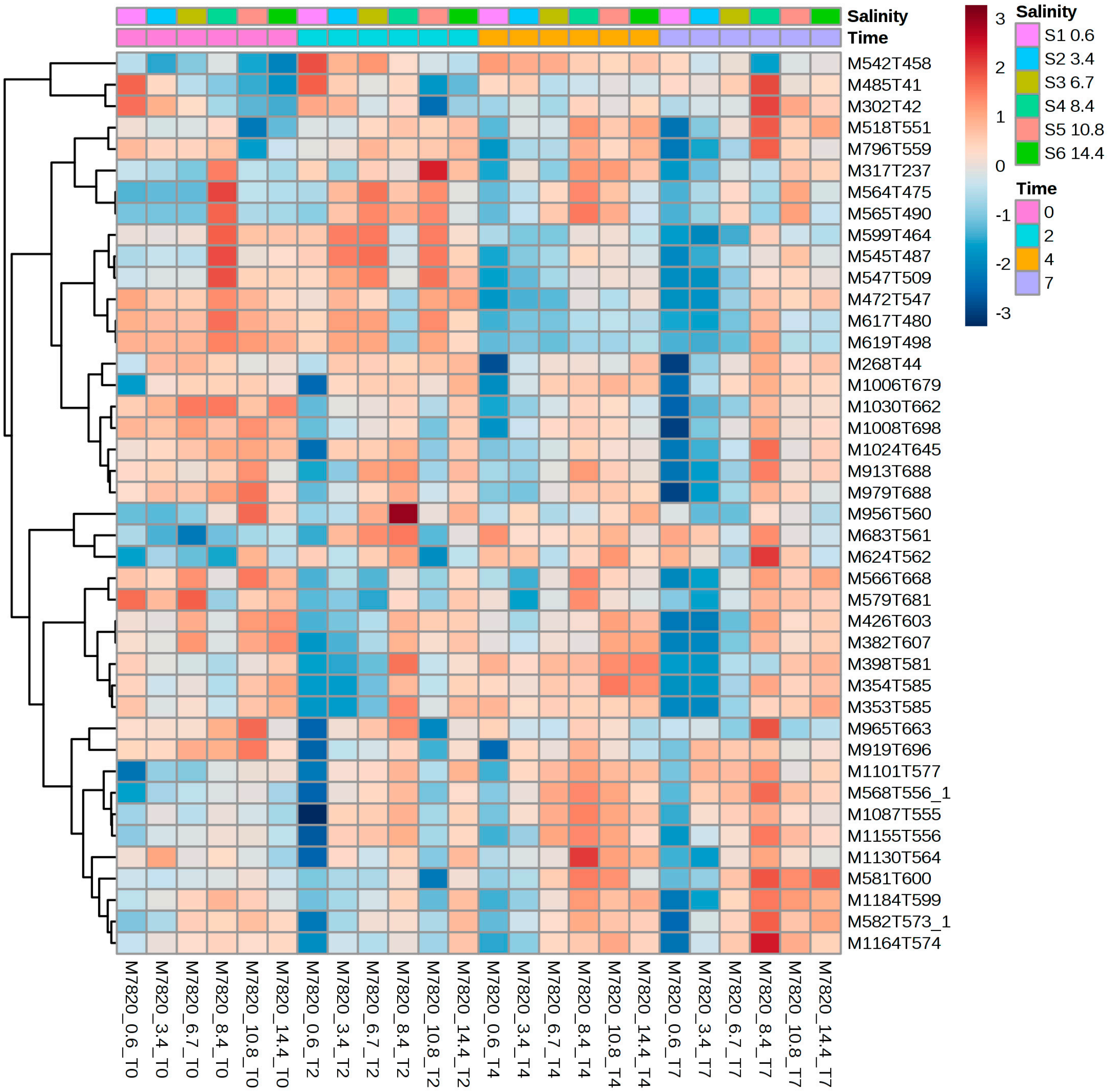

Figure S2. Heatmap of significantly time-affected metabolites of *M. aeruginosa* PCC 7820 (freshwater). Data represented are means of triplicate. (M: mass; T: retention time, time in days) and corresponded to features with a relatively lower (blue) and higher (red) area.

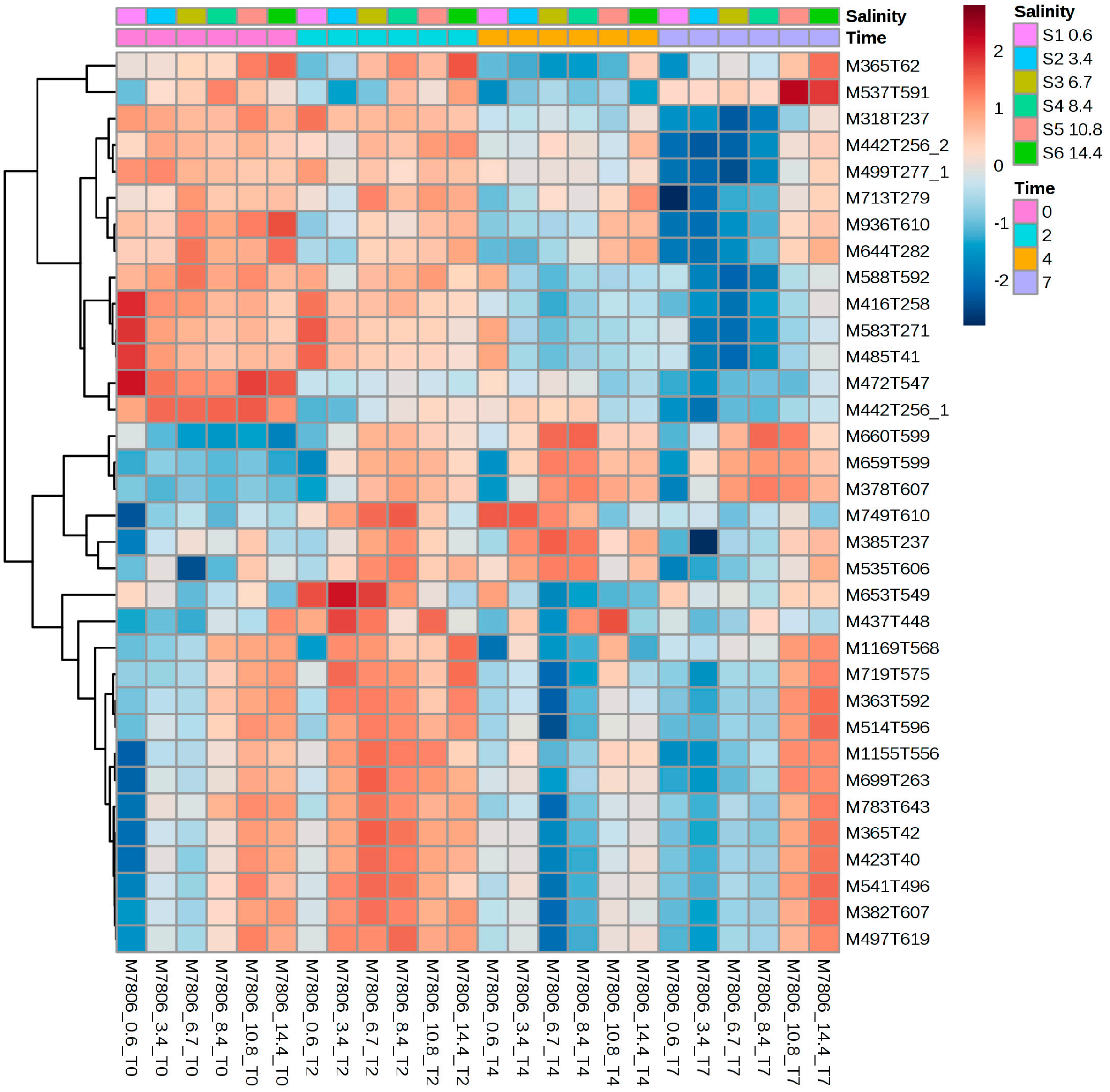

Figure S3. Heatmap of significantly time-affected metabolites of *M. aeruginosa* PCC 7806 (brackish water). Data represented are means of triplicate. (M: mass; T: retention time, time in days) and corresponded to features with a relatively lower (blue) and higher (red) area. Sucrose was extracted in ethanol then evaporated and rehydrated. In order to have a sufficient amount of cells to extract, the cell pellets obtained from the triplicate of cultures were pooled before the extraction (for the salinity 0.6, 6.7, and 10.8). After derivatization, the sucrose was analyzed by gas chromatography coupled to a flame ionization detector (GC-FID) as described by Adams et al. (1999) [73]. Analyses were conducted on a GC system (model HP-6890, Hewlett-Packard) equipped with a CP8008 precolumn (Ret Gap, 2.5 m × 0.32 mm, Agilent Technologies) and a CP-Sil 5 CB capillary column (60 m × 0.32 mm, 0.25 µm, Agilent). Flow rates for air and H_2 for the FID were 400 and 40 mL/min, respectively. Sucrose CAS [57-50-1] (Sigma-Aldrich) was used as standard and myo-inositol as internal standard to determine concentrations.

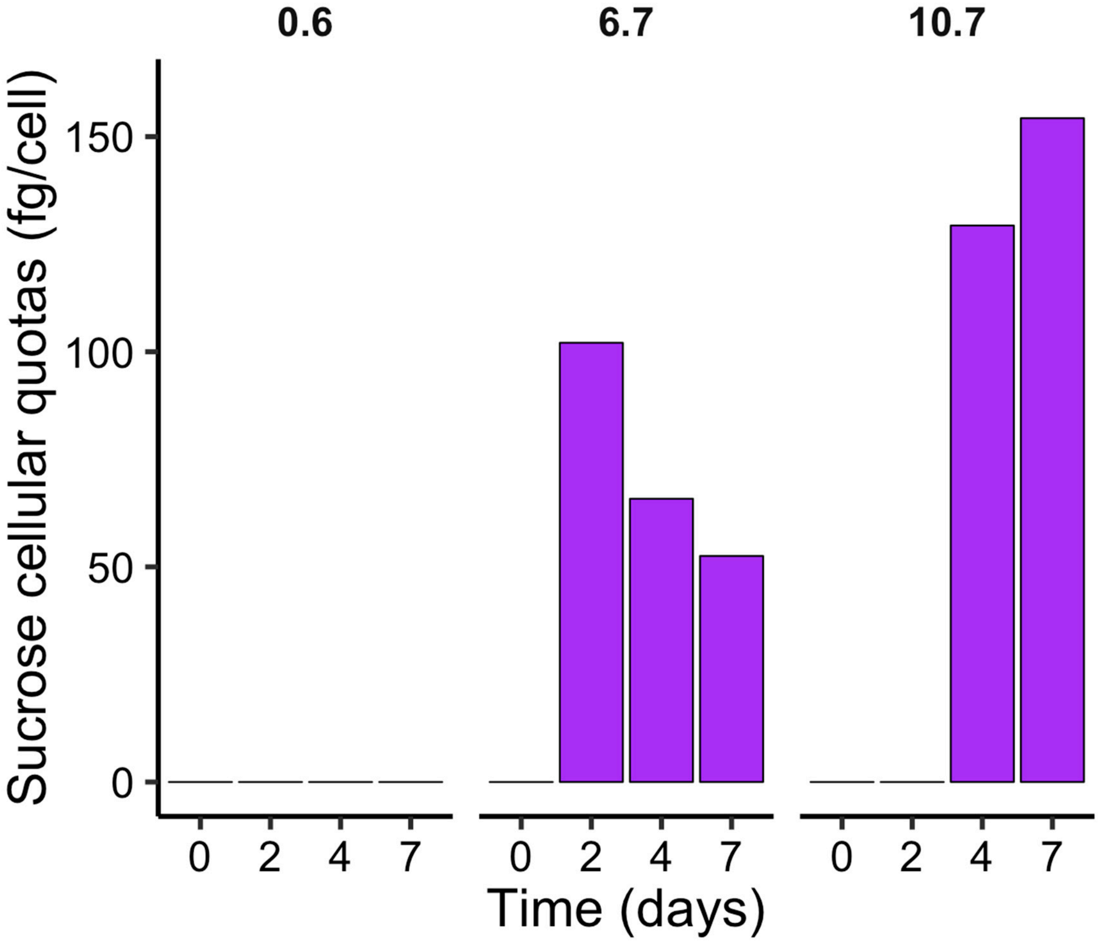

Figure S4. Sucrose cellular quotas over time and salinities for *M. aeruginosa* PCC 7806 (brackish water).

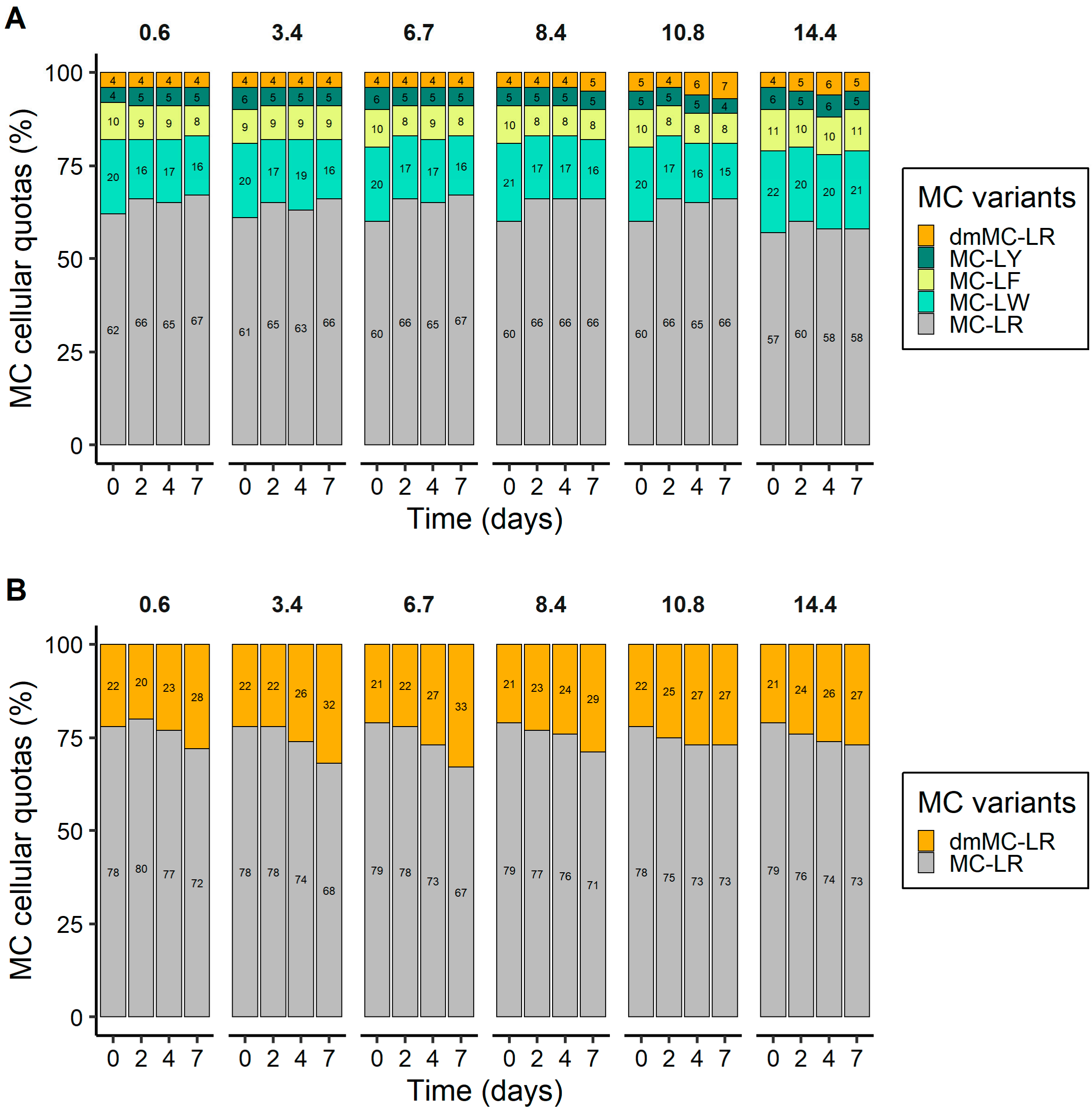

Figure S5. MC cellular quotas over time and salinities expressed as the percentage of each variant relative to the total amount of MCs per cell for *M. aeruginosa* PCC 7820 (freshwater) (**A**) and PCC 7806 (brackish water) (**B**).

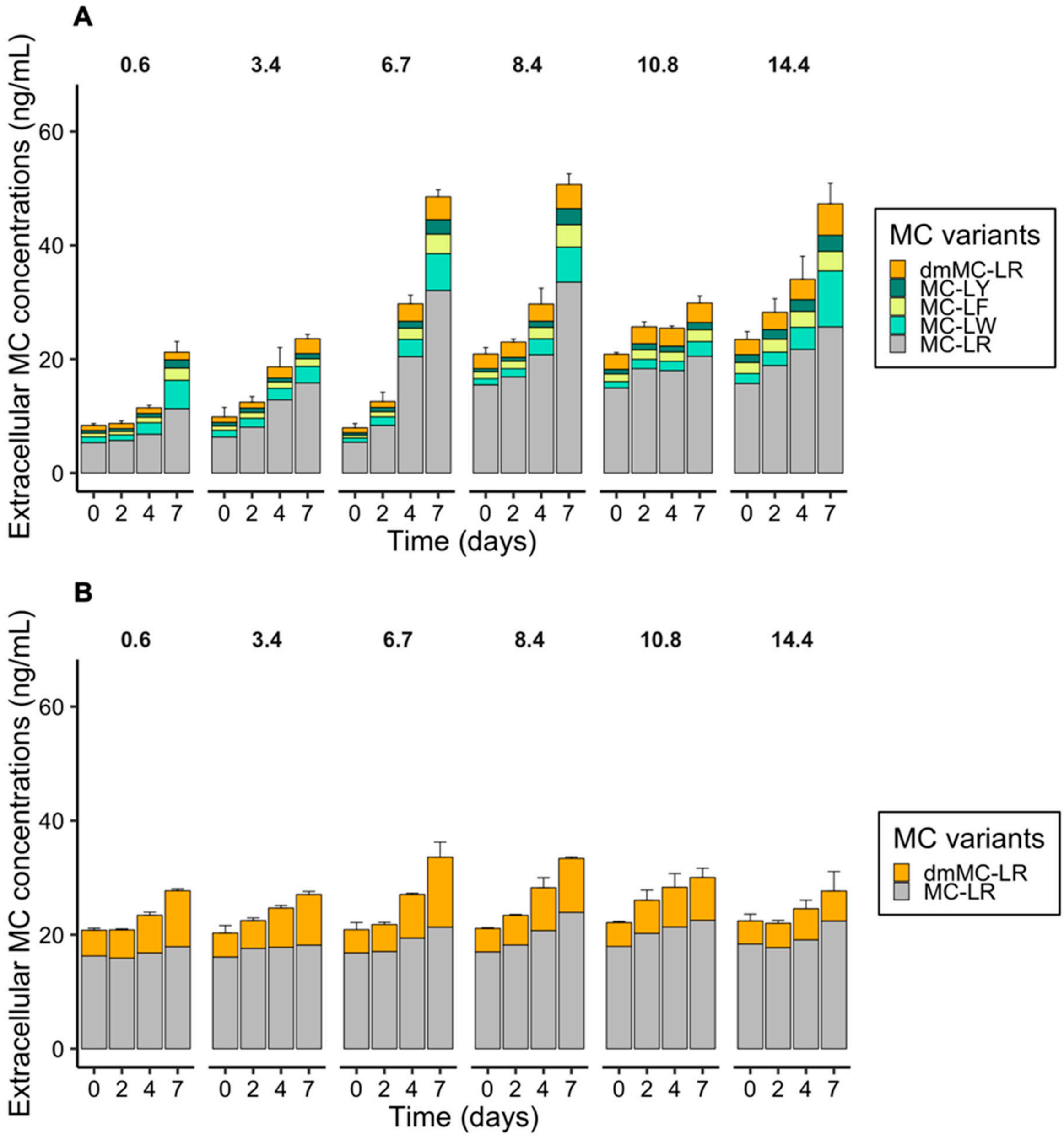

Figure S6. Extracellular MC quotas over time and salinities for *M. aeruginosa* PCC 7820 (freshwater) (**A**) and PCC 7806 (brackish water) (**B**) Error bars represent the standard deviations ($n = 3$).

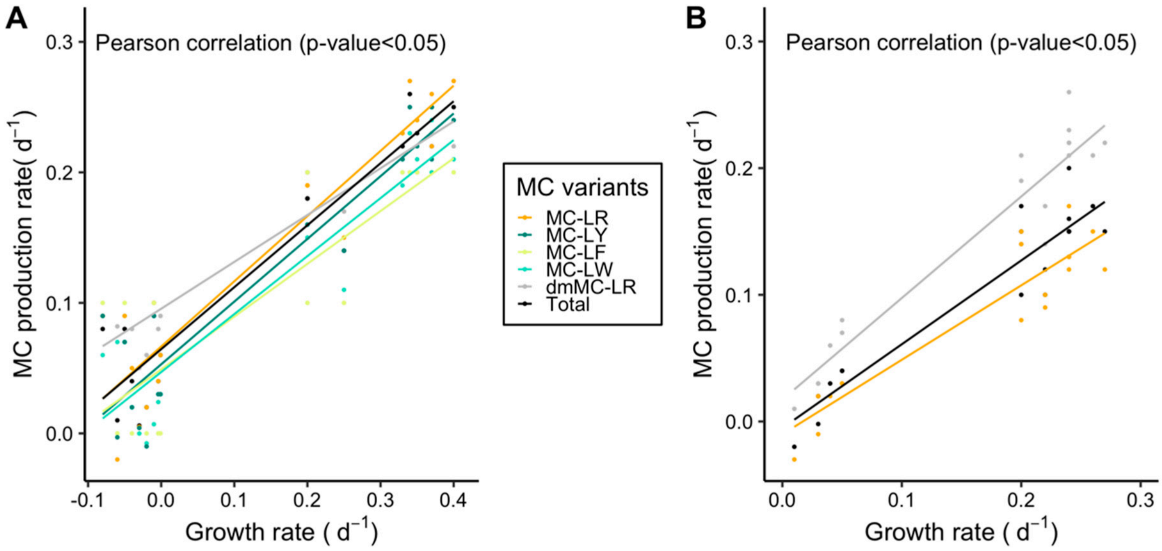

Figure S7. Linear regression between growth rates and MC production rates for *M. aeruginos*a PCC 7820 (freshwater) (**A**) and PCC 7806 (brackish water) (**B**). For both strains and all variants, the Pearson correlation was significant with $p < 0.05$. The "Total" regression curve indicates the linear regression when all the MC variants were pooled to calculate the MC production rate.

Table S1. Primer sequences used for RT-qPCR reactions.

Primer	Forward Sequence (5′ – 3′)	Reverse Sequence (5′ – 3′)	Design
rnpB	TACCTCAAGCGGTTCCTAA	CTGATACCTGATAACTGATTGGG	[61]
sod	AGAAGCATCACCAGCAACCT	GCCTTAGAGCCTTGCATCAC	This study
groEL	CTTGGACCCAAAGGTCGTAA	CCGCTACATCGTTGGTTTTT	This study
psaA	CGGATTTCCTCACCTTCAAA	TTCGAGGATTTCCTTCATGC	This study
psbC	TCTTCGGCTGTACTCGGTTT	GCACCACAACCCAAGAGAAT	This study
spsA	ATCATTCCCTTGCTGCAATC	GCAGATTTTCTGCCTCTTGG	This study
sppA	GTGGGCGATAGTCTGGCTAC	CCGACGGAGGTAATCCAGTA	This study
